# The role of IL22 polymorphisms on liver cirrhosis in patients with hepatitis B virus: A case control study: Erratum

**DOI:** 10.1097/MD.0000000000018477

**Published:** 2019-12-10

In the article, “The role of IL22 polymorphisms on liver cirrhosis in patients with hepatitis B virus: A case control study”,^[1]^ which appeared in Volume 98, Issue 44 of *Medicine*, the incorrect abstract appears in the article. The correct abstract is:

**ABSTRACT**

**AIMS:** Interleukin(IL)-22 plays an important role in promoting liver regeneration and repair, but its role in chronic HBV-related liver diseasesis not clear. The goal of this study was to evaluate associations between eight *IL22* single nucleotide polymorphisms (SNPs) and the development of chronic HBV cirrhosis and HBV-related HCC within a Chinese Han population.

**METHODS:** We investigated associations between single nucleotide polymorphisms (SNPs) in the *IL22* gene (rs1026788, rs2227472, rs2227491, rs2227485, rs1179249, rs2046068,rs2227473, and rs7314777) and the risk of HBV-related chronic liver diseases within a Han population in Northeast China. A total of 649 participants were included in the study, including 103 patients with CHB, 264 patients with LC, and 282 patients with HCC. The odds ratios (OR) and corresponding 95% confidence intervals (CI) were calculated using chi-square test. Haplotype analysis was conducted by haploview software.

**RESULTS:** Genotype and allele distributions of SNPs rs1179249 and rs2227472 differed between LC and CHB groups (both *P* < 0.05).The G alleles of SNP rs2227491 and rs1026788 were more frequent in the LC group than in the CHB group (*P* *=* 0.046, *P* = 0.041 respectively). A *IL22* haplotype consisting of the minor alleles of SNP rs1179249 and the major alleles of seven other SNPs occurred less frequently in the LC and HCC groups than in the CHB group (28.2%, 33.94%, and 37.86%, respectively, *P* < 0.05). Moreover, there were no significant associations between smoking or drinking and *IL22* SNPs on the risk of HCC (*P* > 0.05).

**CONCLUSION:***IL22* genetic variations were associated with chronic HBV infection progression, especially in the HBV-LC group. The *IL22* genetic variations may help clinicians initiate the correct treatment strategy at the CHB stage.
